# Factors Associated With Postpartum Smoking Relapse Among Women Who Quit in Early Pregnancy: The Tohoku Medical Megabank Project Birth and Three-Generation Cohort Study

**DOI:** 10.2188/jea.JE20200609

**Published:** 2023-01-05

**Authors:** Keiko Murakami, Mami Ishikuro, Fumihiko Ueno, Aoi Noda, Tomomi Onuma, Taku Obara, Shinichi Kuriyama

**Affiliations:** 1 Tohoku Medical Megabank Organization, Tohoku University, Sendai, Japan; 2 Graduate School of Medicine, Tohoku University, Sendai, Japan; 3 Department of Pharmaceutical Sciences, Tohoku University Hospital, Sendai, Japan; 4 Department of Disaster Public Health, International Research Institute of Disaster Science, Tohoku University, Sendai, Japan

**Keywords:** early pregnancy, Japan, postpartum smoking relapse, secondhand smoke exposure

## Abstract

**Background:**

While a wide range of predictors of postpartum smoking relapse have been suggested, population-based studies have rarely examined these factors exclusively among women who quit in early pregnancy. Furthermore, workplace secondhand smoke (SHS) exposure has never been examined.

**Methods:**

We analyzed data from 10,466 pregnant women who participated in the Tohoku Medical Megabank Project Birth and Three-Generation Cohort Study. Age, education, parity, breastfeeding, postpartum depression, SHS exposure at home, and SHS exposure at work (not working, working without SHS exposure, working with SHS exposure) were evaluated as possible predictors. Multiple logistic regression analyses were conducted to examine the associations between these factors and smoking relapse by 1 year postpartum among women who quit in early pregnancy. Analyses stratified by SHS exposure at home were also conducted.

**Results:**

About one-fourth of early-pregnancy quitters had relapsed into smoking by 1 year postpartum. Lower education, multiparity, not breastfeeding, postpartum depression, and SHS exposure at home were associated with increased risks of smoking relapse. Working with SHS exposure was associated with an increased risk of smoking relapse; the multivariate-adjusted odds ratios of working without SHS exposure and working with SHS exposure compared with not working were 1.14 (95% confidence interval [CI], 0.82–1.59) and 2.18 (95% CI, 1.37–3.46), respectively. The significant association of workplace SHS exposure was observed only among women without SHS exposure at home.

**Conclusion:**

SHS exposure at work, as well as education, multiparity, breastfeeding, postpartum depression, and SHS exposure at home were associated with postpartum smoking relapse among early-pregnancy quitters.

## INTRODUCTION

Despite decreases in smoking prevalence rates during pregnancy, the relapse rates within the first year postpartum remain high in high-income countries.^1^^,^^2^ Postpartum smoking increases women’s health risks^3^ and exposes infants to secondhand smoke (SHS), which has been linked to sudden infant death syndrome, ear infections, respiratory tract illness, and asthma, as well as deficits in cognitive performance.^4^ Therefore, prevention of postpartum smoking relapse is a public health priority. To design effective interventions for postpartum sustained abstinence, it is important to identify women who are more likely to relapse into smoking after childbirth.

A wide range of predictors of postpartum smoking relapse have been suggested. One systematic review concluded that the most common significant factors were being less well educated; young age; multiparity; living with a partner or household member who smoked; experiencing high stress, depression, or anxiety; not breastfeeding; intending to quit during pregnancy only; and low confidence to remain abstinent postpartum.^5^ Studies examining these factors have mainly evaluated intervention-assisted quitters, postpartum retrospective reports, or reports in middle/late pregnancy.^5^ Women who receive smoking cessation interventions during pregnancy tend to be more nicotine-dependent and thus find it harder to quit.^6^ Retrospective reports on postpartum women are subject to imprecise estimates of when they quit during pregnancy and include women who quit smoking during late pregnancy.^7^ Population-based observational studies have rarely examined these factors exclusively among early-pregnancy quitters,^8^ who quit smoking upon becoming aware of their pregnancy or soon after.^7^^,^^9^ Most of them can maintain cessation throughout their pregnancy, but relapse into smoking during the postpartum period at high rates in Western countries.^9^ Therefore, studies examining factors associated with postpartum smoking relapse among early-pregnancy quitters are also required.

Smoking environment is consistently and strongly associated with postpartum smoking relapse: partner smoking, presence of smokers in the household, not having a smoking ban at home, and high proportion of close associates/family members who are smokers.^5^^,^^10^ Meanwhile, to the best of our knowledge, no studies have examined the association between workplace smoking environment and postpartum smoking relapse, although home and workplace are the two major locations for SHS exposure.^4^ Until recently, smoking in workplaces was not prohibited by law in Japan at the national level.

Considering these circumstances, we aimed to prospectively examine the associations of SHS exposure at work as well as age, education, parity, breastfeeding, postpartum depression, and SHS exposure at home with postpartum smoking relapse among women who quit in early pregnancy in Japan.

## METHODS

### Study population

Data were obtained from the Tohoku Medical Megabank Project Birth and Three-Generation Cohort Study (TMM BirThree Cohort Study), which has been described elsewhere.^11^ Pregnant women and their family members were contacted in obstetric clinics or hospitals when they scheduled their deliveries between 2013 and 2017. Approximately 50 obstetric clinics and hospitals in Miyagi Prefecture participated in the recruitment process. Tohoku University Tohoku Medical Megabank Organization established seven community support centers in Miyagi Prefecture as local facilities for voluntary admission-type recruitment and health assessment of the participants.^12^ Trained genome medical research coordinators were placed in each clinic, hospital, or community support center to provide information on the TMM BirThree Cohort Study to potential participants and to receive a signed informed consent form from each participant. Of 32,968 pregnant women who were contacted, 22,493 agreed to participate. Among them, 587 women were excluded owing to abortion or still birth, withdrawal of participation before identification of birth, and nonidentification of birth statuses. Of the remaining 21,906 women, 11,481 women were excluded: 520 who did not complete the questionnaires in early pregnancy (<14 weeks of gestation), 507 who did not complete the questionnaires in middle pregnancy (14–27 weeks of gestation), 30 whose medical records were not allowed to be transcribed, 8,048 who did not complete the questionnaires at 1 year postpartum, and 2,335 who had missing values for any variables used in the analyses. The remaining 10,466 women were included in the present study. The TMM BirThree Cohort Study protocol was reviewed and approved by the Ethics Committee of Tohoku University Tohoku Medical Megabank Organization (2013-1-103-1). The characteristics of 10,466 included women and 12,027 excluded women are shown in eTable 1.

### Measures

Smoking status was measured at three time points; in early pregnancy, in middle pregnancy, and at 1 year postpartum. In early pregnancy, participants were asked to choose one response option for smoking status: never smoked, quit before becoming aware of pregnancy, quit after becoming aware of pregnancy, and current smoker. In middle pregnancy, participants were asked whether they smoked between early and middle pregnancy. At 1 year postpartum, participants were asked whether they had ever smoked at least 100 cigarettes in their lifetime. If they answered yes, they were additionally asked whether they smoked at that time. Postpartum smoking relapse was defined as smoking at 1 year postpartum among women who had quit smoking after becoming aware of their pregnancy and remained abstinent during pregnancy.^13^

Age in early pregnancy was categorized into three groups: ≤29, 30–34, and ≥35 years. Educational attainment was categorized into three groups: high school or lower (elementary, junior high school, or senior high school), college (2-year college or special training school), and university or higher (university or graduate school). Parity was ascertained from medical records, and dichotomized into nulliparous and multiparous. At 1 year postpartum, participants were asked whether their child had been breastfed. At 1 year postpartum, participants provided responses to the Japanese version of the Edinburgh Postpartum Depression Scale, which comprises ten items assessing any symptoms of depression in the past 7 days.^14^ Each item has four possible responses with scores of 0–3, and the total score ranges from 0–30. Postpartum depression was defined as a score of ≥9.^15^ At 1 year postpartum, participants were asked how often they were exposed to cigarette smoke from someone else at home during the past year. The responses were *almost never or sometimes* and *almost every day* (*≤1 hour/day*, *2–3 hours/day*, *4–5 hours/day*, or *≥6 hours/day*). SHS exposure at home was defined as *almost every day*. SHS exposure at work was measured using a similar question at 1 year postpartum. Work status at 1 year postpartum was dichotomized as working (permanent worker, self-employed worker, temporary worker, or part-time worker) and not working (on leave, pensioner, housewife, student, or unemployed). SHS exposure at work was categorized into three groups: not working, working without SHS exposure, and working with SHS exposure.

### Statistical analysis

Multiple logistic regression analyses were conducted to examine the associations between various factors (age, education, parity, breastfeeding, postpartum depression, SHS exposure at home, and SHS exposure at work) and smoking relapse by 1 year postpartum among women who quit smoking in early pregnancy and remained abstinent during pregnancy. We calculated the odds ratio (OR) and 95% confidence interval (CI) for each factor adjusted for age as well as all other factors. Home and workplace are the two major locations for SHS exposure,^4^ and SHS exposure at home has been previously shown to be associated with the highest risk of postpartum smoking relapse.^5^ Therefore, we also stratified our models by SHS exposure at home, and examined whether this factor modified the associations with smoking relapse by including interaction terms in the models.

All analyses were conducted with SAS version 9.4 software (SAS Institute Inc., Cary, NC, USA). For all analyses, a two-tailed value of *P* < 0.05 was considered statistically significant.

## RESULTS

**Figure 1.**
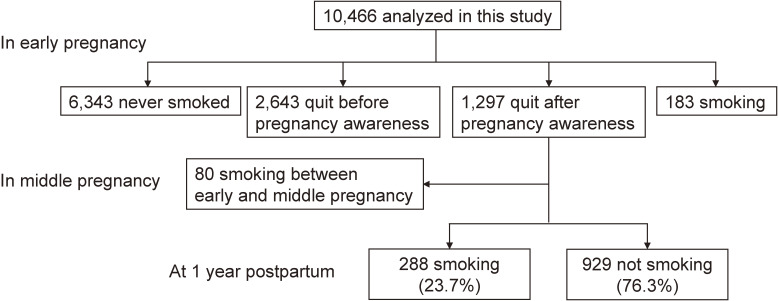
Flow diagram of participants’ smoking status

Table 1 shows the characteristics of the participants. About one-third of them were 29 years old or younger, and had graduated from high school or lower. The prevalence of SHS exposure was 11.1% at home and 5.0% at work. Figure 1 shows a flow diagram of participants’ smoking status. For smoking status in early pregnancy, 6,343 were never smokers (60.6%), 2,643 quit before becoming aware of their pregnancy (25.3%), 1,297 quit after becoming aware of their pregnancy (12.4%), and 183 were current smokers (1.7%). Among 1,297 women who quit in early pregnancy, 80 relapsed into smoking by middle pregnancy. Among women who remained abstinent during pregnancy, 23.7% had relapsed into smoking by 1 year postpartum.

**Table 1.** Characteristics of participants: the Tohoku Medical Megabank Project Birth and Three-Generation Cohort Study (*n* = 10,466)

	*n*	(%)
Age, years		
≤29	3,434	(32.8)
30–34	3,987	(38.1)
≥35	3,045	(29.1)
Educational attainment		
University or higher	3,029	(28.9)
College	4,076	(39.0)
High school or lower	3,361	(32.1)
Parity		
Nulliparous	5,173	(49.4)
Multiparous	5,293	(50.6)
Breastfeeding		
No	4,381	(41.9)
Yes	6,085	(58.1)
Postpartum depression		
No	9,120	(87.1)
Yes	1,346	(12.9)
SHS exposure at home		
No	9,305	(88.9)
Yes	1,161	(11.1)
SHS exposure at work		
Not working	4,767	(45.5)
Working without SHS exposure	5,176	(49.5)
Working with SHS exposure	523	(5.0)

SHS, secondhand smoke.

Table 2 presents the prevalences, ORs, and 95% CIs for smoking relapse by 1 year postpartum among women who quit in early pregnancy. Age was not associated with postpartum relapse; the multivariate-adjusted OR of age ≥35 years compared with age ≤29 years was 0.74 (95% CI, 0.49–1.10). Lower education was associated with an increased risk of postpartum relapse; the multivariate-adjusted OR of ≤high school education compared with ≥university education was 1.95 (95% CI, 1.12–3.40). Multiparity was associated with an increased risk of postpartum relapse (multivariate-adjusted OR 2.37; 95% CI, 1.74–3.23). Breastfeeding was associated with a decreased risk of postpartum relapse (multivariate-adjusted OR 0.18; 95% CI, 0.13–0.26). Postpartum depression was associated with an increased risk of postpartum relapse (multivariate-adjusted OR 1.51; 95% CI, 1.04–2.19). SHS exposure at home was associated with an increased risk of postpartum relapse (multivariate-adjusted OR 2.53; 95% CI, 1.83–3.49). Working without SHS exposure was not associated with postpartum relapse, while working with SHS exposure was associated with an increased risk of postpartum relapse; the multivariate-adjusted ORs of working without SHS exposure and working with SHS exposure compared with not working were 1.14 (95% CI, 0.82–1.59) and 2.20 (95% CI, 1.37–3.46), respectively.

**Table 2.** Prevalence, OR and 95% CI for smoking relapse by 1 year postpartum among women who quit in early pregnancy (*n* = 1,217)

	Smoking relapse/ early-pregnancy quitters	(%)	Age-adjusted		Multivariate-adjusted^a^	
			OR (95% CI)		OR (95% CI)	
Age, years						
≤29	154/546	(28.2)	1.00		1.00	
30–34	80/395	(20.3)	0.65	(0.48–0.88)	0.75	(0.52–1.06)
≥35	54/276	(19.6)	0.62	(0.44–0.88)	0.74	(0.49–1.10)
Educational attainment						
University or higher	20/150	(13.3)	1.00		1.00	
College	79/439	(18.0)	1.45	(0.85–2.46)	1.25	(0.70–2.24)
High school or lower	189/628	(30.1)	2.70	(1.63–4.46)	1.95	(1.12–3.40)
Parity						
Nulliparous	111/667	(16.6)	1.00		1.00	
Multiparous	177/550	(32.2)	2.57	(1.95–3.39)	2.37	(1.74–3.23)
Breastfeeding						
No	238/657	(36.2)	1.00		1.00	
Yes	50/560	(8.9)	0.18	(0.13–0.25)	0.18	(0.13–0.26)
Postpartum depression						
No	223/1,007	(22.1)	1.00		1.00	
Yes	65/210	(31.0)	1.56	(1.12–2.16)	1.51	(1.04–2.19)
SHS exposure at home						
No	158/914	(17.3)	1.00		1.00	
Yes	130/303	(42.9)	3.49	(2.62–4.65)	2.53	(1.83–3.49)
SHS exposure at work						
Not working	112/562	(19.9)	1.00		1.00	
Working without SHS exposure	114/526	(21.7)	1.15	(0.86–1.55)	1.14	(0.82–1.59)
Working with SHS exposure	62/129	(48.1)	3.68	(2.45–5.52)	2.18	(1.37–3.46)

CI, confidence interval; OR, odds ratio; SHS, secondhand smoke.

^a^Adjusted for all other variables in the table.

Table 3 presents the results of the analyses stratified by SHS exposure at home. For age, education, parity, and postpartum depression, interactions by SHS exposure at home were not detected. Breastfeeding was associated with postpartum relapse among both SHS exposure at home groups, although an interaction was detected (*P* = 0.041). Working with SHS exposure was significantly associated with postpartum relapse only among women without SHS exposure at home; the multivariate-adjusted ORs of working with SHS exposure compared with not working were 2.94 (95% CI, 1.52–5.67) among women without SHS exposure at home and 1.65 (95% CI, 0.86–3.22) among women with SHS exposure at home.

**Table 3.** Prevalence, OR and 95% CI for smoking relapse by 1 year postpartum among women who quit in early pregnancy according to SHS exposure at home (*n* = 1,217)

	No SHS exposure at home (*n* = 914)				SHS exposure at home (*n* = 303)				*P* for interaction^b^
	
	Smoking relapse/ early-pregnancy quitters	(%)	Multivariate-adjusted^a^		Smoking relapse/ early-pregnancy quitters	(%)	Multivariate-adjusted^a^		
			OR (95% CI)				OR (95% CI)		
Age, years									0.20
≤29	75/386	(19.4)	1.00		79/160	(49.4)	1.00		
30–34	45/305	(14.8)	0.82	(0.52–1.28)	35/90	(38.9)	0.68	(0.38–1.21)	
≥35	38/223	(17.0)	0.87	(0.54–1.40)	16/53	(30.2)	0.48	(0.23–1.02)	
Educational attainment									0.96
University or higher	13/128	(10.2)	1.00		7/22	(31.8)	1.00		
College	48/353	(13.6)	1.27	(0.64–2.53)	31/86	(36.1)	1.31	(0.43–3.95)	
High school or lower	97/433	(22.4)	2.03	(1.04–3.94)	92/195	(47.2)	1.83	(0.65–5.18)	
Parity									0.49
Nulliparous	65/533	(12.2)	1.00		46/134	(34.3)	1.00		
Multiparous	93/381	(24.4)	2.54	(1.72–3.76)	84/169	(49.7)	2.05	(1.22–3.43)	
Breastfeeding									0.041
No	134/462	(29.0)	1.00		104/195	(53.3)	1.00		
Yes	24/452	(5.3)	0.14	(0.09–0.22)	26/108	(24.1)	0.29	(0.17–0.51)	
Postpartum depression									
No	126/771	(16.3)	1.00		97/236	(41.1)	1.00		0.86
Yes	32/143	(22.4)	1.52	(0.93–2.46)	33/67	(49.3)	1.44	(0.79–2.63)	
SHS exposure at work									
Not working	57/429	(13.0)	1.00		55/133	(41.4)	1.00		0.063
Working without SHS exposure	79/425	(18.6)	1.40	(0.93–2.11)	35/101	(34.7)	0.76	(0.42–1.35)	
Working with SHS exposure	22/60	(36.7)	2.94	(1.53–5.67)	40/69	(58.0)	1.65	(0.86–3.17)	

CI, confidence interval; OR, odds ratio; SHS, secondhand smoke.

^a^Adjusted for all other variables in the table.

^b^Interaction between each factor and SHS exposure at home.

## DISCUSSION

The present study examined factors associated with postpartum smoking relapse among women who quit in early pregnancy in Japan. About one-fourth of early-pregnancy quitters had relapsed into smoking by 1 year postpartum. Lower education, multiparity, not breastfeeding, postpartum depression, and SHS exposure at home were associated with increased risks of postpartum smoking relapse. Working with SHS exposure was associated with an increased risk of postpartum smoking relapse, especially among women without SHS exposure at home, while working without SHS exposure was not associated with postpartum smoking relapse.

Among women who quit in early pregnancy, 23.7% had relapsed into smoking by 1 year postpartum. One systematic review reported that 43% of intervention-assisted quitters had relapsed by 6 months postpartum.^6^ In a cross-sectional study conducted in 2009 using a nationally representative sample in Japan, 43.4% of postpartum women who quit smoking at any point during pregnancy retrospectively reported that they had relapsed into smoking by 18 months postpartum.^16^ Meanwhile, a prospective study conducted from 2014 to 2015 in Nagoya, Japan found that 24.2% of women who quit in early pregnancy had relapsed into smoking by 18 months postpartum.^8^ It has been suggested that women who quit smoking upon becoming aware of their pregnancy or soon after are more likely to maintain long-term postpartum abstinence than intervention-assisted quitters^10^^,^^17^ and those who only achieve abstinence in late pregnancy.^13^^,^^18^^,^^19^ In Japan, the smoking prevalence rates during pregnancy and within the child-rearing period have been gradually declining in recent years, partly through the implementation of various programs against smoking during pregnancy.^20^ These situations can explain the lower prevalence of postpartum smoking relapse among Japanese women who quit in early pregnancy.

Lower education, multiparity, not breastfeeding, and postpartum depression were associated with increased risks of postpartum smoking relapse. These factors were consistently shown to be associated with postpartum smoking relapse among intervention-assisted quitters and quitters at some point during pregnancy.^5^ The present study also demonstrated these associations among women who quit in early pregnancy. The smoking status of early-pregnancy quitters, like that of pre-pregnancy quitters, is often ignored at obstetric visits, and these women receive little or no help to support and maintain cessation during pregnancy or to prevent relapse in the postpartum period.^9^ Our findings for the common factors associated with postpartum smoking relapse suggest that similar relapse prevention interventions would be helpful for both early-pregnancy quitters and other quitters.

SHS exposure at home was associated with an increased risk of postpartum smoking relapse. The association between home smoking environment and postpartum relapse was consistent with previous findings.^5^^,^^10^ The influence of being around other smokers on relapse was shown to be multifaceted, encompassing behavioral influence, peer pressure, ready access to cigarettes, and positive associations with smell and taste.^21^^,^^22^ There has been a growing recognition of the need to include partners and other household members in interventions directed at smoking cessation and SHS exposure reduction among pregnant women.^23^^,^^24^ A comprehensive intervention that encourages partners and other household members to quit smoking or avoid SHS^10^ and empowers women to manage their SHS exposure within the home^25^ could result in a significant reduction in postpartum relapse rates.

The present study also showed an association between workplace SHS exposure and postpartum smoking relapse, with a stronger association among women without SHS exposure at home than among women with SHS exposure at home. This finding indicates that not only the home environment but also the workplace environment are important for postpartum smoking relapse. The workplace is an important space for and influence on smoking behaviors.^26^ Many adults spend the majority of their day in a workplace environment, and the workplace has the potential for reinforcing social support networks and peer influences among coworkers.^27^ There is sufficient evidence that smoke-free policies decrease tobacco use when implemented in the workplace in the general population.^28^^,^^29^ In Japan, there was no national legislation prohibiting indoor smoking^30^; the Health Promotion Act allowed for partial bans as an option, and the Workplace Smoke-free Guideline recommended a partial rather than a complete ban in 2003. The Industrial Safety and Health Act, which was revised in 2015, asks for appropriate management to prevent workplace SHS exposure, but does not mandate a complete smoking ban. However, the revised Health Promotion Act that prohibits smoking in public facilities has been implemented in stages and came into full force in April 2020. This workplace improvement can be expected to decrease the postpartum smoking relapse rate.

The present findings have implications for the design of effective interventions aimed at reducing postpartum smoking relapse. Women are more likely to quit smoking during pregnancy than at any other time of their life, because of their knowledge about fetal effects and social pressure not to smoke during pregnancy.^31^ Women who quit upon becoming aware of their pregnancy are often highly motivated to protect their fetuses, which is an external and temporary motivator.^9^ Once this motivation disappears after childbirth, they can relapse into smoking.^22^ However, the health risks of smoking for infants continue after childbirth; infants whose parents are smokers are more likely to be exposed to SHS^32^ and to eventually become smokers themselves.^33^ Additionally, most pregnant women are relatively young and healthy, and quitting smoking earlier in life is associated with greater health benefits than quitting later in life.^34^ It is necessary to shift motivational considerations from an almost exclusive focus on fetal protection to a wider consideration of quitting for the health and well-being of infants and mothers. Smoking cessation interventions are effective in supporting pregnant women to quit,^35^^,^^36^ but there is little evidence that interventions for postpartum smoking relapse are effective in the long-term postpartum period.^17^^,^^37^ The postpartum period is a window of opportunity to maintain smoking cessation among women who have successfully quit during pregnancy. The present study has revealed factors that can be used to identify high-risk subpopulations of pregnant women and can be targeted in future relapse-prevention interventions.

The present study has several limitations. First, we were only able to analyze about half of the pregnant women who agreed to participate in the TMM BirThree Cohort Study. The women who were excluded from the analysis were less well educated, had greater prevalence of postpartum depression, had higher exposure to SHS, and were more likely to be smokers than the women who were included in the analysis (eTable 1). Second, the study was conducted in 1 of the 47 prefectures in Japan, so the generalizability of the present findings is limited. A national survey reported that the percentages of smoking during pregnancy were 3.8% in 2013 and 2.7% in 2017,^20^ which was higher than 1.8% noted in the present study. However, the previous measures for assessing smoking were retrospective reports from mothers during the childrearing period, which were different from our measures. Third, smoking status was self-reported, which is a source of uncertainty because women may be influenced by social desirability, a bias that tends to be important when questions deal with socially undesirable attitudes and behaviors.^38^ Confirmation of abstinence is best achieved when self-reported measures are validated with biomarkers, such as cotinine levels, although these data were not available in the present study. Finally, data on smoking status in late pregnancy were not obtained. Postpartum smoking relapse defined in the present study might include smoking relapse in late pregnancy, although early-pregnancy quitters are shown to typically remain abstinent throughout the pregnancy.^9^ Data on the details of quitting smoking were also not obtained. Information on the intention and way to quit smoking will be helpful for a better understanding of the mechanism of postpartum smoking relapse.

In conclusion, about one-quarter of women who quit in early pregnancy had relapsed into smoking by 1 year postpartum. Lower education, multiparity, not breastfeeding, postpartum depression, and SHS exposure at home were associated with increased risks of postpartum smoking relapse. Working with SHS exposure was associated with an increased risk of postpartum smoking relapse, especially among women without SHS exposure at home. These factors should be considered when designing interventions to prevent postpartum smoking relapse, which can improve maternal and child health.

## SUPPLEMENTARY MATERIAL

eTable 1. Characteristics differences between 10,466 women who were analyzed and 12,027 women who were excluded from the analysis
