# Supplementary material for: Factors Associated With Postpartum Smoking Relapse Among Women Who Quit in Early Pregnancy: The Tohoku Medical Megabank Project Birth and Three-Generation Cohort Study
Source: J Epidemiol. 2023 Jan 5;33(1):8–14. doi: 10.2188/jea.JE20200609 (PMC9727209; doi:10.2188/jea.JE20200609)
Supplement: Supplementary file 1 [file je-33-008-s001.pdf]

eTable 1. Characteristics differences between 10,466 women who were analyzed and 12,027 women who were excluded from the analysis

	Women who were analyzed (n=10,466)		Women who were not analyzed (n=12,027)		*P*-value[a]
	n	(%)	n	(%)	
Age, years					<0.001
≤29	3,434	(32.8)	4,260	(39.0)	
30–34	3,987	(38.1)	3,809	(34.9)	
≥35	3,045	(29.1)	2,851	(26.1)	
Educational attainment					0.0012
University or higher	3,029	(28.9)	813	(26.3)	
College	4,076	(39.0)	1,183	(38.3)	
High school or lower	3,361	(32.1)	1,091	(35.4)	
Parity					0.035
Nulliparous	5,173	(49.4)	5,612	(48.0)	
Multiparous	5,293	(50.6)	6,077	(52.0)	
Breastfeeding					0.12
No	4,381	(41.9)	1,008	(40.2)	
Yes	6,085	(58.1)	1,502	(59.8)	
Postpartum depression					0.025
No	9,120	(87.1)	2,263	(85.5)	
Yes	1,346	(12.9)	384	(14.5)	
SHS exposure at home					<0.001
No	9,305	(88.9)	1,313	(81.2)	
Yes	1,161	(11.1)	304	(18.8)	
SHS exposure at work					0.0012
Not working	4,767	(45.5)	431	(43.7)	
Working without SHS exposure	5,176	(49.5)	479	(48.6)	
Working with SHS exposure	523	(5.0)	76	(7.7)	
Smoking in early pregnancy					<0.001
Never smoked	6,343	(60.6)	6,381	(58.9)	
Quit before pregnancy awareness	2,643	(25.3)	2,321	(21.4)	
Quit after pregnancy awareness	1,297	(12.4)	1,773	(16.4)	
Smoking	183	(1.8)	356	(3.3)	
Smoking at 1 year postpartum					0.0058
No	9,937	(95.0)	2,887	(93.7)	
Yes	529	(5.0)	195	(6.3)	

SHS, secondhand smoke.
Percentages are shown after excluding women whose values were missing.
[a]Obtained using the chi-square test, comparing women who were analyzed and women who were not.
